# Supplementary material for: Reduction in Five Harmful Substances in Fried Potato Chips by Pre-Soaking Treatment with Different Tea Extracts
Source: Foods. 2023 Jan 9;12(2):321. doi: 10.3390/foods12020321 (PMC9858103; doi:10.3390/foods12020321)
Supplement: Supplementary file 1 [file foods-12-00321-s001.zip › foods-2095775-supplementary.pdf]

Reduction of five harmful substances in fried potato chips by pre-soaking treatment with different tea extracts

Weitao Wang [1,†], Huaixu Wang [1,†], Zhongjun Wu [2], Tingting Duan [3], Pengzhan Liu [4], Shiyi Ou [2,5], Hani El-Nezami [1,6,*] and Jie Zheng [2,5,*]

Table S1. Mass spectrometric parameters for SRM analysis of catechins in different teas.

Analyte	Operate mode	Precursor ion	S-lens (eV)	Fragment ion	Collision energy (eV)	Retention time (min)
C	Positive	290.700	53	139.016*	15	4.98
				147.043	19	
ECG	Positive	443.026	55	122.974*	24	6.24
				138.990	42	
EGC	Positive	307.018	54	139.036*	15	4.74
				162.967	19	
EGCG	Positive	459.035	52	138.999*	26	5.33
				150.923	16	
EC	Negative	289.000	62	245.049*	18	5.57
				203.029	21	
CG	Negative	440.950	77	168.967*	22	6.42
				289.064	20	
GC	Negative	305.000	64	125.040*	22	3.37
				219.058	19	
GCG	Negative	457.007	79	168.997*	19	5.50
				124.974	44	

* Represents the fragment ion for quantification.
